# Conflict-related health research in Syria, 2011–2019: a scoping review for *The Lancet* - AUB Commission on Syria

**DOI:** 10.1186/s13031-021-00384-3

**Published:** 2021-12-14

**Authors:** Marian Abouzeid, Manal K. Elzalabany, Iman Nuwayhid, Samer Jabbour

**Affiliations:** 1 The Lancet-American University of Beirut Commission on Syria, Faculty of Health Sciences, American University of Beirut, Beirut, Lebanon; 2 Alfred Deakin Institute for Citizenship and Globalisation and Centre for Humanitarian Leadership, Deakin University, Burwood, Australia; 3 Faculty of Health Sciences, American University of Beirut, P.O.Box: 11-0236, Riad El Solh, Beirut, 1107 2020 Lebanon

## Abstract

**Background:**

The volume of health-related publications on Syria has increased considerably over the course of the conflict compared with the pre-war period. This increase is largely attributed to commentaries, news reports and editorials rather than research publications. This paper seeks to characterise the conflict-related population and humanitarian health and health systems research focused inside Syria and published over the course of the Syrian conflict.

**Methods:**

As part of a broader scoping review covering English, Arabic and French literature on health and Syria published from 01 January 2011 to 31 December 2019 and indexed in seven citation databases (PubMed, Medline (OVID), CINAHL Complete, Global Health, EMBASE, Web of Science, Scopus), we analyzed conflict-related research papers focused on health issues inside Syria and on Syrians or residents of Syria. We classified research articles based on the major thematic areas studied. We abstracted bibliometric information, study characteristics, research focus, funding statements and key limitations and challenges of conducting research as described by the study authors. To gain additional insights, we examined, separately, non-research publications reporting field and operational activities as well as personal reflections and narrative accounts of first-hand experiences inside Syria.

**Results:**

Of 2073 papers identified in the scoping review, 710 (34%) exclusively focus on health issues of Syrians or residents inside Syria, of which 350 (49%) are conflict-related, including 89 (25%) research papers. Annual volume of research increased over time, from one publication in 2013 to 26 publications in 2018 and 29 in 2019. Damascus was the most frequently studied governorate (*n* = 33), followed by Aleppo (*n* = 25). Papers used a wide range of research methodologies, predominantly quantitative (*n* = 68). The country of institutional affiliation(s) of first and last authors are predominantly Syria (*n* = 30, 21 respectively), the United States (*n* = 25, 19 respectively) or the United Kingdom (*n* = 12, 10 respectively). The majority of authors had academic institutional affiliations. The most frequently examined themes were health status, the health system and humanitarian assistance, response or needs (*n* = 38, 34, 26 respectively). Authors described a range of contextual, methodological and administrative challenges in conducting research on health inside Syria. Thirty-one publications presented field and operational activities and eight publications were reflections or first-hand personal accounts of experiences inside Syria.

**Conclusions:**

Despite a growing volume of research publications examining population and humanitarian health and health systems issues inside conflict-ravaged Syria, there are considerable geographic and thematic gaps, including limited research on several key pillars of the health system such as governance, financing and medical products; issues such as injury epidemiology and non-communicable disease burden; the situation in the north-east and south of Syria; and besieged areas and populations. Recognising the myriad of complexities of researching active conflict settings, it is essential that research in/on Syria continues, in order to build the evidence base, understand critical health issues, identify knowledge gaps and inform the research agenda to address the needs of the people of Syria following a decade of conflict.

**Supplementary Information:**

The online version contains supplementary material available at 10.1186/s13031-021-00384-3.

## Introduction

The armed conflict in Syria is commonly described as being among the most extensively studied and documented of contemporary conflicts [1]. There is an expanding volume of literature on a diverse range of health issues relating to the conflict, from attacks on healthcare and use of chemical weapons to refugee health status assessment. Research in and on active conflict zones is inherently difficult, and these challenges are reflected in the focus, nature and volume of research outputs produced while an armed conflict is ongoing. For example, while the challenges facing the health systems of countries hosting large numbers of Syrian refugees have been documented [2, 3], research assessments of the fragmented health system(s) inside war-torn Syria are limited. Similarly, compared with populations inside Syria, Syrian refugee populations are more accessible and therefore have received comparatively more research attention, including reviews and syntheses of this large volume of published research [4].

*The Lancet* - American University of Beirut Commission on Syria is an international research collaboration launched in December 2016 to analyse the Syrian conflict, its toll and the international response through a health and wellbeing lens, and to propose a set of recommendations to address current and future needs, inform rebuilding efforts and drive accountability [5]. To inform its work, the Commission has conducted a number of literature reviews. A paper examining clinical, biomedical, public health and health systems articles on Syria published between 1991 and 2017 reported that compared with the pre-conflict period (i.e. pre-2011), over the course of the conflict the number of health-related publications increased while the rate, type, topic and local authorship of publications changed. News, commentaries and editorial publications and not research largely drove the increase in publication volume during the conflict period [6]. Whilst non-research publications are crucial to raise awareness, rapidly disseminate information and inform advocacy during active conflicts, research in/on active war zones is also essential. To the best of our knowledge, a detailed thematic overview of conflict-related research on health inside Syria has not been published. Such a review is important to understand what themes, population subgroups and geographic areas have been examined, allowing identification of knowledge gaps to inform the health research agenda. In this paper, we aim to characterise conflict-related population and humanitarian health and health systems research focused inside Syria and published over the course of the conflict.

## Methods

### Search strategy

This study is a sub-analysis of a broader scoping review of literature published between 2011 and 2019 and examining human health and Syria or Syrians, including studies of refugees and multi-country publications that include but are not exclusively limited to Syria. This review is different to, and complements, the prior review by Abdul-Khalek et al. [6] in that it covers a larger set of issues, has a broader geographical scope (inside Syria, outside Syria, multicountry settings including Syria), covers exclusively the conflict years and over a longer time-span of the conflict, and has specifically examined publications by conflict and non-conflict-related status. The search strategy for the broader scoping review is provided in the Additional file 1. Briefly, the broader scoping review searched for literature on human health and Syria or Syrians published in English, Arabic or French from 1 January 2011 to 31 December 2019 and indexed in seven bibliographic and citation databases (PubMed, Medline (OVID), CINAHL Complete, Global Health, EMBASE, Web of Science, Scopus). After de-duplication, 13,699 records were identified. Two-stage screening against pre-specified criteria by at least two reviewers, review of reference lists and review of additional papers known to the authors resulted in a total of 2073 relevant publications.

For this current analysis, we examined only conflict-related research papers that studied health issues inside Syria and focused on Syrians or residents in Syria (e.g. Palestinian or Iraqi refugees). We defined conflict-related publications as those examining the Syrian conflict, its effects or the conflict response. We defined research papers as publications of any type (including traditional research papers, letters, commentaries and other) that report on the primary collection of data, or the secondary analysis and interpretation of existing data. We therefore excluded review publications and literature syntheses, as well as non-conflict related publications (e.g. biomedical or genomic studies conducted during the war but not related to the Syrian conflict, its effects or the conflict response), clinical case studies and case series, conflict-related studies focused only on Syrians outside Syria (e.g. studies of Syrian refugees or studies of war casualties evacuated for treatment in neighbouring countries), and studies conducted in multiple countries that include Syria as one of any number of study countries. Field and operational activities publications, defined as papers describing operational activities or organizational field experiences inside Syria but not presenting research per se (e.g. papers describing set-up of a field facility and number of patients seen, or describing humanitarian operations), which provide important insights or data about health issues inside Syria during the conflict, were considered separately to the research papers. Similarly, personal reflections and narrative accounts of first-hand experiences inside Syria were considered separately. News reports and news interviews with personnel inside Syria were not included in this analysis.

### Literature dataset analysis

We classified research articles into six categories based on the major thematic areas studied: mortality; population health status; health determinants and risks (including behavioural, physiological, environmental and social determinants of health); humanitarian assistance, response or needs (including any studies conducted by humanitarian agencies or analysis of services provided by humanitarian actors); health system (including papers examining any of the six health system pillars as defined by the World Health Organization [7], namely service delivery (unless delivered by humanitarian actors), health workforce, health information systems, medical products, financing, governance); and war strategies & alleged international humanitarian law (IHL) violations (including studies reporting on warfare, besiegement and related human rights violations, attacks on civilian infrastructures such as health facilities, and publications on chemical attacks). A single paper could be classified into multiple thematic categories if it had a major focus on more than one theme.

For each paper, at least one reviewer abstracted bibliometric information and study characteristics (including study description, study period, methodology, governorates/ geographic location of the study, study population, country of affiliation(s) of first and last authors, type of institutional affiliation(s) of first and last authors). We also extracted qualitative data on selected key limitations and challenges of conducting research as described by the authors, and categorized these as contextual (which we defined as including issues of safety, accessibility, stakeholder engagement and cultural considerations), methodological (including issues related to design and conduct of the study) or administrative (including issues related to research permits and permissions, logistics, research capacity). Where available, funding statements were also reviewed and assigned to one of three categories (funded, not funded, not reported). Classification of each paper was discussed by three reviewers.

We used basic descriptive statistics to summarise key bibliometric characteristics of the research papers and changes in the volume and focus of research over time. Key challenges encountered were summarized narratively.

## Results

Among the 13,699 records initially identified through the full scoping review, 2073 papers were considered relevant to human health and Syria. For this conflict-related research subanalysis, we excluded clinical case reports and case series (*n* = 169), publications that examined multiple countries of which Syria was one (*n* = 268), publications that studied Syrians outside Syria (*n* = 924), publications focussed inside Syria but not related to the conflict (*n* = 359) and conflict-related publications focussed inside Syria that were not research publications, personal reflections or descriptions of field and operational activities (*n* = 225). This resulted in a total of 89 conflict-related research publications focused inside Syria, which form the dataset of this analysis. These conflict-related research papers were all published in English. We also identified 31 English-language field and operational activities papers focused on health inside Syria and eight personal narrative reflections, which we examine separately.

**Fig. 1:**
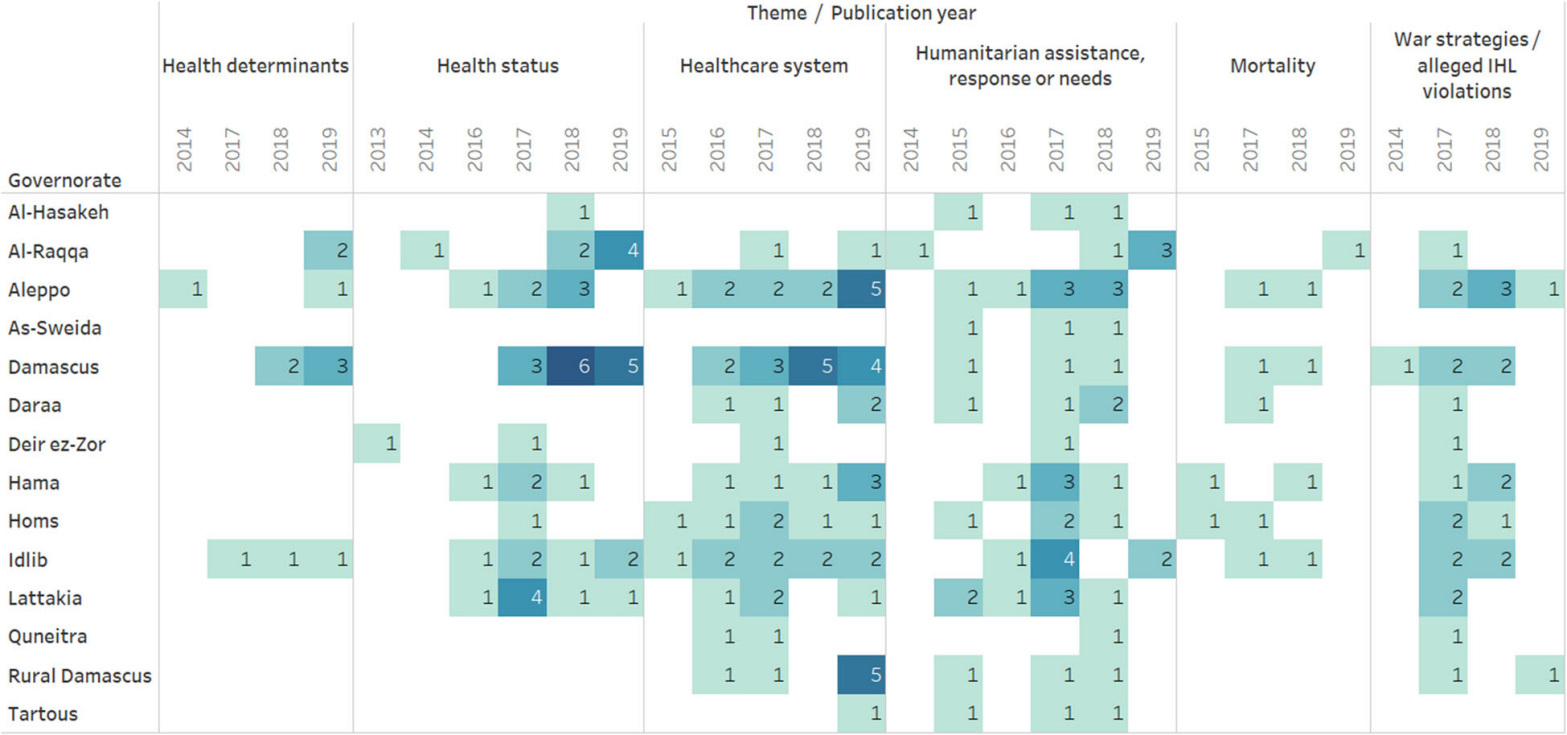
Governorates examined in research publications on health inside Syria by themes, January 2011–December 2019

Table 1 presents summary characteristics of the 89 conflict-related research papers. The conflict in Syria started in 2011 but there were no conflict-related research papers published during 2011–2012. Thereafter the annual volume of research increased over time, from one publication in 2013, three in 2014, to 26 publications in 2018 and 29 in 2019. There is considerable variation in the governorates studied by thematic focus and over time (Table 1, Fig. 1). Damascus is the most frequently studied governorate (*n* = 33), followed by Aleppo (*n* = 25), Idlib (*n* = 20), Lattakia (*n* = 15) and Hama (*n* = 14). Deir Al Zour (*n* = 3), Quenietra (*n* = 3) and As-Sweida (*n* = 3) are the least frequently studied. Twelve papers have a national scope. Several papers do not identify specific governorates, instead referring to the controlling factions, describing for example opposition-controlled territories generally. There are no papers on health in areas while controlled by the so-called Islamic State (IS).

**Table 1:** Characteristics of conflict-related research studies on health inside Syria by research theme, January 2011–December 2019

	Total number of research publications	THEME^a^					
		Health status	Mortality	Health determinants and risks	Humanitarian assistance, response or needs	Health system	War strategies & alleged IHL violations
**Publication year**							
2011–2013	1	1	0	0	0	0	0
2014–2016	18	6	2	1	6	8	2
2017–2019	70	31	8	11	20	26	12
**Location**^b^							
***By governorate***							
Al-Hasakeh	4	1	0	0	3	0	0
Al-Raqqa	8	7	1	2	5	2	1
Aleppo	25	6	2	2	8	12	6
As-Sweida	3	0	0	0	3	0	0
Damascus	33	14	2	5	3	14	5
Daraa	9	0	1	0	4	4	1
Deir ez-Zor	3	2	0	0	1	1	1
Hama	14	4	2	0	5	6	3
Homs	12	1	2	0	4	6	3
Idlib	20	6	2	3	7	9	4
Lattakia	15	7	0	0	7	4	2
Quneitra	3	0	0	0	1	2	1
Rural Damascus	10	0	0	0	3	7	2
Tartous	4	0	0	0	3	1	0
***By region***^g^							
National/ whole of Syria	12	6	3	0	1	8	4
Border with Turkey/ North-west Syria	3	0	0	0	3	1	0
Non-government-controlled areas (governorates not specified)	1	0	1	0	0	0	1
Opposition controlled areas (governorates not specified)	4	1	1	0	3	1	1
Not reported	3	0	1	0	1	1	1
**First author’s country of institutional affiliation** ^e^							
Syria	30	21	1	6	3	12	0
USA	25	8	3	3	9	11	6
UK	12	3	1	2	4	6	2
Belgium	6	1	3	0	3	0	3
Lebanon	3	0	1	0	0	2	2
Turkey	3	0	0	0	2	1	1
Other^c^	15	11	1	1	5	4	1
Not stated	2	0	0	1	1	1	0
**First author’s institutional affiliation**^f^							
Academic	69	26	7	9	15	27	13
Clinical	7	5	0	1	2	3	0
Governmental	1	1	0	0	0	1	0
Humanitarian^h^	14	10	3	3	10	2	0
Independant	1	0	0	1	0	1	0
Military	1	0	0	0	0	0	1
Think tank/ research organization	2	2	0	1	0	1	0
United Nations agency	2	1	0	0	0	2	1
**Senior (last) author’s country of institutional affiliation** ^**e**^							
Syria	21	15	0	5	0	7	0
USA	19	3	5	1	3	10	8
UK	10	3	2	2	5	5	1
Lebanon	7	3	2	0	0	4	2
Belgium	5	2	0	0	5	0	0
Turkey	5	0	0	1	4	1	1
Other^d^	24	15	1	2	8	10	3
Not applicable	4	1	0	1	1	1	1
Not stated	1	1	0	0	1	0	0
**Senior (last) author’s institutional affiliation**^f^							
Academic	65	29	8	8	13	26	11
Clinical	7	3	0	0	1	4	2
Governmental	2	2	0	0	0	0	0
Humanitarian^h^	13	8	2	3	9	3	0
Independant	2	0	0	0	2	0	0
Military	1	0	0	0	0	0	1
United Nations agency	2	0	0	0	1	1	1
Not applicable	4	1	0	1	1	1	1
**Methodology**							
Primary quantitative	40	20	1	9	13	13	2
Secondary quantitative	28	15	9	0	5	11	7
Qualitative	15	2	0	2	4	7	4
Mixed methods	6	1	0	1	4	3	1
**Research funding**							
Funded	37	10	5	7	14	11	7
Not funded	28	17	4	4	7	10	5
Not reported	24	11	1	1	5	13	2

^a^ Some studies cover multiple themes

^b^ Due to the fluid nature of the conflict and inconsistencies in reporting of study locations, we report here the governorates whenever they are reported by the study authors, regardless of political control (i.e. opposition-controlled, government-controlled or non-government-controlled). When the governorates are not specified but the political control of the study setting is reported by study authors, it is recorded as such in this table. Some studies report neither the political control nor the governorates, and these are denoted as ‘not reported’. Some studies cover multiple governorates

^c^ Other includes Australia (1), Austria (1), Egypt (2), Israel (1), Japan (1), Qatar (2), Saudi Arabia (2), Spain (2), Switzerland (1), The Netherlands (2)

^d^ Other includes Australia (3), Austria (1), Canada (2), Egypt (1), France (3), Germany (1), India (1), Israel (1), Jordan (2), Qatar (1), Saudi Arabia (2), Singapore (1), Sweden (1), The Netherlands (4)

^e^ Six publications have first authors with multiple affiliations, while seven publications have multiple last author affiliations. Not applicable means there is no senior author (i.e. single author publication). Not stated means there is an author but their affiliation is not mentioned

^f^ Academic institutional affiliations include universities and university-affiliated clinical facilities

^g^One study examined remote cross-border operations from the Turkish border city of Gaziantep

^h^The humanitarian category denotes humanitarian operational or other nongovernmental organisations

Papers have used a wide range of research methodologies, including primary quantitative methods (*n* = 40) such as surveys, questionnaires and clinical trials; secondary quantitative data analysis (*n* = 28) mainly using surveillance system, medical record or program data; and qualitative methodologies (*n* = 15). Six papers used mixed methods (Table 1).

For the majority of papers, the country of institutional affiliation(s) of first and last (assumed to be the senior) authors are Syria (*n* = 30, 21 respectively), the United States (*n* = 25, 19 respectively) or the United Kingdom (*n* = 12, 10 respectively). For 20 papers (22.4%), both first and last authors had a Syrian affiliation. The institutional affiliation of first authors was predominantly academic (including universities and university hospitals) (*n* = 69), followed by humanitarian organizations (*n* = 14), clinical facilities (*n* = 7), think tank or research organization (*n* = 2), United Nations (UN) agencies (*n* = 2), government (*n* = 1), military (*n* = 1) and independent (*n* = 1). The institutional affiliation of senior/ last authors was predominantly academic (*n* = 65), followed by humanitarian (*n* = 13) or clinical (*n* = 7) organisations, with a few senior authors affiliated to governmental or UN agencies (*n* = 2 for each), and military (*n* = 1). Of the 37 papers reporting a specific funding source, five listed Syrian universities as the funding source.

### Research themes

Table 2 presents detailed summaries of each research paper.

**Table 2:** Summary of conflict-related health research studies inside Syria, January 2011–December 2019

First author (Publication year)	First & Last (Senior) authors’ country of institutional affiliation	Type of institutional affiliation	Description	Theme (Subtheme)	Study Period (month/year)	Methodology	Study Population	Geographic focus	Funding
**Alasaad (2013)** [8]	First: Syria, Switzerland, Spain Last: N/A	First: academic	Reviews YouTube and social media videos to describe leishmaniasis outbreaks in Deir ez-Zor.	Health status	January–March 2013	YouTube and Facebook video review, contact with local communities	General population	Deir ez-Zor	Not reported
**Ahmad (2014)** [9]	First: UK Last: N/A	First: academic	Assesses the influences of neighborhood socioeconomic position and urban informal settlements on women’s health.	Health determinants and risks	April–May 2011	Key informant interviews, unstructured observations	Professionals working with populations in informal communities; women in informal settlements	Aleppo	Institute of Health and Society, Newcastle University, UK
**Hoetjes (2014)** [10]	First: Netherlands Last: Netherlands	First: humanitarian Last: humanitarian	Documents results of a screening program and examines food security and factors affecting nutritional status amongst the Tal-Abyad population (Al-Raqqa), and describes MSF nutrition programs.	Health status; humanitarian assistance, response or needs	2013	Mixed methods (middle-upper arm circumference screening; qualitative survey)	IDPs living in schools	Al-Raqqa	Not reported
**Rosman (2014)** [11]	First: Israel Last: Israel	First: academic/ military Last: academic/ military	Reports on physician reviews of YouTube videos documenting a sarin attack to determine clinical presentation and review the management of a mass casualty event	War strategies / alleged IHL violations	August–September 2013	Physician review of YouTube videos	Casualties of Ghouta sarin attack	Damascus	Not reported
**Alshiekhly (2015)** [12]	First: Syria Last: Syria	First: academic Last: academic	Evaluates feasibility of Facebook as a teaching modality for a course on medical emergencies in dental practice	Health system (workforce)	March–April 2014	Pre and post questionnaire	Dental students	Not reported	None
**Doocy (2015)** [13]	First: USA Last: Belgium	First: academic Last: academic	Documents humanitarian needs and priorities among households in need of assistance in accessible communities	Humanitarian assistance, response or needs	April–June 2014	Needs assessment	General population	Predominantly government-controlled areas (Aleppo, As-Sweida, Damascus, Dara’a, Al-Hasakeh, Homs, Lattakia, Rural Damascus, Tartous)	Humanitarian assistance programs
**Doocy (2015)** [14]	First: USA Last: USA	First: academic Last: academic	Describes trends in internal displacement and analyzes the association between displacement and household well-being and humanitarian needs.	Humanitarian assistance, response or needs	March 2011–June 2014	Review of published IDP estimates; needs assessment	General population	National	Not reported
**Guha-Sapir (2015)** [15]	First: Belgium Last: Lebanon	First: academic Last: academic	Documents direct conflict-related deaths of women and children using VDC data.	Mortality; war strategies / alleged IHL violations	March 2011–January 2015	VDC data analysis	Civilians	National	emBRACE—Building resilience amongst communities in Europe & Chatham House
**Price (2015)** [16]	First: USA Last: USA	First: humanitarian Last: humanitarian	Applies capture - recapture methods to data from four sources to estimate mortality, and describes the issues associated with accurately documenting mortality estimates in conflict settings	Mortality	December 2012–March 2013	Multiple systems estimation / capture -recapture	Civilians and combatants	Homs, Hama	Not reported
**Sekkarie (2015)** [17]	First: USA Last: USA	First: clinical Last: academic	Explores the impact of conflict on dialysis services	Health system (service provision, workforce)	2013	Key informant Interviews	Dialysis facility administrators, providers and patients	Non-government-controlled areas (Aleppo, Homs, Idlib)	Not reported
**Tajaldin (2015)** [18]	First: USA Last: USA	First: academic Last: academic	Compares polio case rates using laboratory versus clinical case definitions and different surveillance systems	Health system (information systems); health status	2013–2014	Analysis of EWARS and EWARN data	General population	National	Not reported
**Trelles (2015)** [19]	First: Belgium Last: Belgium	First: humanitarian Last: humanitarian	Reports on surgical cases and intraoperative mortality rates at an MSF field hospital	Humanitarian assistance, response or needs	September 2012–January 2014	Analysis of MSF programme data	Surgical patients	Jabal Al Akrad, Latakia Northwest Syria	Médecins Sans Frontières-Operational Centre, Belgium.
**Charlson (2016)** [20]	First: Australia, USA Last: Australia, USA	First: academic Last: academic	Estimates prevalence of depression and PTSD and models likely current and future mental health service requirements	Health status; Health system (service provision, workforce)	Global Burden of Disease Study 2010, modelling for period 2015–2030	Global Burden of Disease (GBD) methodology and modelling	General population	National	Queensland Department of Health, Australia
**El-Khani (2016)** [21]	First: UK Last: UK	First: academic Last: academic	Examines feasibility of the “bread wrapper approach” which involves distribution of psychological support information on leaflets inserted into bread wraps and using the bread wraps to circulate questionnaires assessing the usefulness of the information provided.	Humanitarian assistance, response or needs	Not reported	Questionnaire	General population	Northern Syria next to the borders with Turkey	The University of Manchester’s ESRC Transformative Research Prize Committee
**Elsafti (2016)** [22]	First: Qatar, Egypt Last: Belgium	First: clinical Last: academic	Documents the family, educational, and health status of Syrian children and associated humanitarian needs	Health status; humanitarian assistance, response or needs	May 2015	Needs assessment	Children less than 15 years old	Aleppo, Hama, Idlib, Lattakia	None
**Ismail (2016)** [23]	First: UK Last: Lebanon	First: academic Last: academic	Describes trends in tuberculosis, measles and polio case numbers in Syria and challenges of disease surveillance in the conflict context.	Health system (information systems); health status	Peer-reviewed analysis (2005–2015), EWARN & EWARS analysis (2014–2015)	Analysis of EWARN, EWARS data	General population	National	Not reported
**Jefee-Bahloul (2016)** [24]	First: USA Last: USA	First: academic Last: academic	Examines attitudes of Syrian healthcare providers towards Store & Forward tele-mental health consultations	Health system (service provision)	Not reported	Online survey	Syrian health-care professionals affiliated with humanitarian NGOs	Aleppo, Damascus, Idlib, Others^a^	Not reported
**Mowafi (2016)** [25]	First: USA Last: France	First: academic Last: humanitarian	Assesses the functional status and capacity of trauma hospitals	Health system (service provision, workforce)	February–March 2015	Survey	Hospitals providing secondary or tertiary surgical care in non-government-controlled areas	Aleppo, Damascus (non-government-controlled area), Dara’a, Hama, Homs, Idlib, Lattakia, Quneitra, Rural Damascus	Yale University & UOSSM.
**Sparrow (2016)** [26]	First: USA Last: Australia	First: academic Last: academic	Compares two infectious disease surveillance systems, the Ministry of Health-run EWARS system and the independent, non-governmental EWARN	Health system (information system); health status	2014–2015	Analysis of EWARS and EWARN data	General population	National	Not reported
**Al-Saadi (2017)** [27]	First: Syria Last: Syria	First: academic Last: academic	Reports prevalence and risk factors of psychological distress among medical students in Damascus	Health status; Health system (workforce)	November 2015	Online questionnaire	Second to sixth-year medical students at Damascus University	Damascus	None
**Alsaied (2017)** [28]	First: USA Last: Canada, Qatar	First: clinical Last: academic, clinical	Reports on the operations of UOSSM^b^ primary care clinics in Opposition Territories and reasons for consultations	Humanitarian assistance, response or needs; health status	January 2014–December 2015	Retrospective administrative database review	Patients seen at 10 primary care centers	Opposition-controlled areas (Aleppo, Deir ez-Zor, Hama, Homs, Idlib, Lattakia)	None
**Arafat (2017)** [29]	First: Syria Last: Syria	First: clinical Last: academic	Describes differences in patterns of injury and mortality rates among patients with penetrating abdominal injuries at Damascus Hospital	Health status	October 2012–June 2013	Retrospective review of records	Patients with penetrating abdominal injuries	Damascus	None
**Baaity (2017)** [30]	First: Syria Last: Syria	First: academic Last: academic	Reports the prevalence of extended spectrum β lactamases of *E. coli* in isolates from patients in Al-Assad Teaching Hospital, Lattakia	Health status	October 2014–November 2016	Analysis of data regarding clinical isolates	Patients in Al-Assad teaching hospital	Lattakia	Not reported
**Cummins (2017)** [31]	First: Not stated Last: Turkey	First: humanitarian Last: humanitarian	Aims to understand the impact of providing food kits in Idlib and how it disturbs the food market, and proposes market-based approach as an alternative for food aid provision.	Humanitarian assistance, response or needs	2016	Interviews	Community members in Idlib (Darkoush and Salquin)	Idlib	UK Department for International Development (DFID) through the Urban Crises Learning Fund.
**Diggle (2017)** [32]	First: UK Last: UK	First: humanitarian Last: academic	Reflects on operational experiences and analyses humanitarian health response data collected in contested and opposition-held areas of Syria	Mortality; health status; humanitarian assistance, response or needs	2013–2014	Analysis and review of data from multiple operational databases	Civilians & combatants	Contested and opposition-controlled areas (governorates not specified)	None
**Doocy (2017)** [33]	First: USA Last: USA	First: academic Last: academic	Identifies humanitarian needs and priorities among displaced and female headed households in government-controlled areas	Humanitarian assistance, response or needs	April–June 2016	Needs assessment	Displaced & female headed households	Government-controlled areas (Aleppo, As-Sweida, Damascus, Dara’a, Hama, Al-Hasakeh, Homs, Lattakia, Rural Damascus, Tartous)	US-based international nongovernmental organization
**Doocy (2017)** [34]	First: USA Last: Turkey	First: academic Last: humanitarian	Examines the effectiveness of three assistance programs (in-kind food commodities, food vouchers, unrestricted vouchers) in improving food security in northern Syria.	Humanitarian assistance, response or needs; Health determinants and risks	September–December 2014, May/June 2015	Serial household surveys, shopkeeper survey and analysis of program monitoring data	Beneficiary households; shopkeepers participating in the voucher program	Idlib	GOAL
**Elamein (2017)** [35]	First: Turkey Last: Turkey, Egypt	First: UN agency Last: UN agency, academic	Describes the first operational use of the Monitoring Violence against Health Care (MVH) tool to report real-time incident data for attacks on healthcare infrastructure, workers and patients	War strategies / alleged IHL violations; health system (service provision; workforce)	November 2015–December 2016	Analysis of MVH data	Healthcare workers, health service users	Aleppo, Al-Raqqa, Damascus, Dara’a, Deir ez-Zor, Hama, Homs, Idlib, Lattakia, Quneitra, Rural Damascus	None
**Fouad (2017)** [36]	First: Lebanon Last: Lebanon	First: academic Last: academic	Reports on attacks on healthcare and experiences of health workers inside Syria	War strategies / alleged IHL violations; health system (workforce; service provision)	Not reported	Mixed methods study (quantitative data analysis, consultations, testimonials)	Health workers	National	IDRC; American University of Beirut
**Fujita (2017)** [37]	First: Japan Last: Sweden	First: academic Last: academic	Presents daily time series analysis of violent deaths in Syria, including temporal and spatial analysis, and compares trends with those of violent and non-violent deaths in the non-conflict context of England	Mortality	1200-day period commencing 500 days post conflict onset	Secondary analysis and modelling of Violations Documentation Center data	Civilians and military personnel	Aleppo, Damascus, Dara’a, Homs, Idlib	Bilateral Joint Research Project between JSPS, Japan, and FRS-FNRS, Belgium. Grants-in-Aid for Scientific Research from MEXT Japan
**Hawat (2017)** [38]	First: Syria Last: Syria	First: academic Last: academic	Reviews the epidemiology of cutaneous and visceral leishmaniasis in Lattakia and examines the effects of the Syrian conflict on incidence	Health status	2006–2016	Analysis of Leishmaniasis and Contagious Diseases Centre data for new cases of leishmaniasis	General population	Lattakia	Not reported
**Mohammad (2017)** [39]	First: Syria Last: Canada	First: academic Last: clinical, academic	Assesses asthma prevalence, asthma control and quality of life among those with diagnosed asthma; Among non-asthmatics, estimates prevalence of respiratory symptoms, PTSD symptoms and other chronic disease co-morbidities.	Health status	Not reported	Cross-sectional survey	IDPs in Al-Herjalleh shelter aged > = 5 years	Damascus	Syrian Private University
**Sahloul (2017)** [40]	First: USA Last: USA	First: academic Last: clinical	Examines quality of cancer care and needs in government-controlled areas versus besieged areas, and provision of care in general clinics compared with specialized cancer care clinics.	Health system (service provision, workforce); war strategies / alleged IHL violations	Not reported	Cross-sectional survey	Oncologists and surgeons working in cancer clinics, and general physicians	Government-controlled areas (Damascus, Lattakia, Homs, and West Aleppo) and besieged areas (East Ghouta, East Aleppo, and Idlib)	Not reported
**van Berlaer (2017)** [41]	First: Belgium Last: Belgium	First: academic Last: academic	Documents diagnoses, injuries and comorbidities in children in Northern Syria	Health status; humanitarian assistance, response or needs	May 2015	Cross-sectional household survey	Children younger than 15 years	Aleppo, Hama, Idlib, Lattakia	None
**Abbas (2018)** [42]	First: Syria Last: Syria	First: academic Last: academic	Examines efficacy and feasibility of peer- led versus professional-led training in basic life support course for medical students	Health system (workforce)	April 2016	Randomized controlled trial	Medical students in pre-clinical years at Syrian Private University	Damascus	None
**Albaroudi (2018)** [43]	First: Syria Last: Syria	First: academic Last: academic	Examines prevalence of iron deficiency and iron deficiency anemia in children and socioeconomic associations, and effectiveness of oral iron supplements	Health status; health determinants and risks	1) Retrospective part: November 2011–November 2015 2) Prospective part: 2 month period, not specified	Retrospective medical record review; parental questionnaire & clinical data analysis	Children seen at primary care clinics at the Children’s Hospital in Damascus	Damascus	Damascus University
**Ballouk (2018)** [44]	First: Syria Last: Syria	First: academic Last: academic, government	Assesses gingival health status in children aged 8–12 years in Damascus city	Health status	September 2016–January 2017	Cross-sectional, school-based oral health survey	Children aged 8–12 years	Damascus	None
**Chen (2018)** [45]	First: USA Last: USA	First: academic Last: academic	Estimates the number of unique identifiable deaths in the Syrian conflict by deduplicating four datasets	Mortality; war strategies and alleged IHL violations	March 2011–April 2014	Unique entity estimation using mortality data from VDC, SNHR, CSR-SY, SS^c^	Civilians and combatants	Not reported	National Science Foundation (NSF), Amazon Research Award, Laboratory for Analytic Sciences (LAS).
**Darwish (2018)** [46]	First: Syria Last: Syria	First: academic Last: academic	Reviews pediatric chest injuries treated in the Mouassat University Hospital in Damascus before and during the Syrian crisis	Health status	January 2005–December 2016	Hospital record review	Pediatric chest trauma patients	Damascus	None
**de Lima Pereira (2018)** [47]	First: Syria Last: Netherlands	First: humanitarian Last: humanitarian	Assesses vaccine-preventable disease risk and vaccination needs, and vaccination coverage following an immunization program	Humanitarian assistance, response or needs; health status	June–September 2015	Cross-sectional household survey	Children < 5 years old	Aleppo	None
**Doocy (2018)** [48]	First: USA Last: USA	First: academic Last: academic	Assesses humanitarian needs in government-controlled areas of Syria	Humanitarian assistance, response or needs	April – June 2016	Needs assessment	General population	Government-controlled areas (Aleppo, As-Sweida, Damascus, Dara’a, Hama, Al-Hasakeh, Homs, Lattakia, Rural Damascus, Tartous)	US-based international nongovernmental organization
**Footer (2018)** [49]	First: USA Last: USA	First: academic Last: academic	Explores the impact of the Syrian conflict on health workers and healthcare in opposition-controlled areas and challenges faced, including in responding to chemical weapons attacks	Health system (service provision, workforce); war strategies / alleged IHL violation	October 2014 (Gaziantep), July–August 2017 (online)	Semi-structured interviews	Health-care professionals with experience in opposition-controlled areas of Syria	Opposition- controlled areas (governorates not specified)	MacArthur Foundation
**Garry (2018)** [50]	First: UK Last: UK	First: academic Last: academic	Explores factors impacting provision of healthcare for NCDs in opposition-controlled Syria	Humanitarian assistance, response or needs	June–August 2017	Semi-structured interviews	Humanitarian healthcare staff or Syrian health workers from opposition-controlled / contested areas	Opposition-controlled areas (governorates not specified)	UK Research and Innovation (Global Challenges Research Fund)
**Guha-Sapir (2018)** [51]	First: Belgium Last: USA	First: academic Last: academic	Reports demographic, spatial, and temporal patterns of direct deaths among civilians and opposition combatants using VDC data	Mortality; war strategies / alleged IHL violations	March 2011–December 2016	VDC data analysis	Civilians and combatants	Non-government-controlled areas (governorates not specified)	None
**Haar (2018)** [52]	First: USA Last: USA	First: academic Last: academic	Describes a standardized field survey tool for documenting attacks on healthcare, and compares this dataset to PHR’s database that uses open sources to track attacks on health facilities	War strategies / alleged IHL violations; health system (service provision, workforce)	2016	Development of prospective surveillance methodology, standardized reporting questionnaire	NA	Aleppo, Hama, Homs, Idlib	MacArthur Foundation, the Oak Foundation, Berkeley Research Impact Initiative
**Idris (2018)** [53]	First: Syria Last: Syria	First: academic Last: academic	Reports prevalence of cigarette smoking among university students and examines the impact of war on smoking behavior	Health determinants and risks	May 2015	Online cross-sectional survey	Undergraduate students at Damascus University.	Damascus	None
**Khamis (2018)** [54]	First: Syria Last: Lebanon	First: government Last: academic	Reports National AIDS Program data and examines how the war affected HIV surveillance and voluntary counselling and testing	Health status; health system (service provision)	2010–2016	Secondary analysis of National AIDS Program surveillance data	General population	National	Not reported
**Kubitary (2018)** [55]	First: Syria Last: Syria, France	First: academic Last: academic	Examines validity of the Arabic version of the two-question Quick Inventory of Depression (QID-2-Ar) in Syrian multiple sclerosis patients.	Health status	Not reported	Cross-sectional study	Multiple sclerosis patients aged 18–60 years seen at two hospitals	Damascus	None
**Kubitary (2018)** [56]	First: Syria Last: France, Syria	First: academic Last: academic	Examines the effects of Therapy by Repeating Phrases of Positive Thoughts (TRPPT) on PTSD, Sleep Disorder and War Experiences among school children and adolescents	Health systems (service provision); health status	Not reported	Clinical trial	Children & adolescents aged 13–17 years	Damascus	Not reported
**Meiqari (2018)** [57]	First: Netherlands Last: Netherlands	First: humanitarian, academic Last: humanitarian	Describes the impact of the war on child health in Tal Al-Abyad and Kobane, using available medical and humanitarian data from Médecins Sans Frontières	Health status; humanitarian assessment, response or needs	April 2013–September 2016	Analysis of MSF clinical data and reports	Children < 5 years old who attended an MSF facility	Aleppo, Al-Raqqa	Not reported
**Morrison (2018)** [58]	First: UK Last: N/A	First: academic	Reports the experiences and challenges of health service delivery under siege	Health system (service provision); war strategies / alleged IHL violations	Mid-2016	Interviews and focus group discussions	Interim Council and healthcare professionals in four besieged urban areas	Opposition-controlled areas (Aleppo, Damascus)	DFID’s Urban Crises Programme
**Othman (2018)** [59]	First: Not stated Last: Australia	First: independent Last: academic	Evaluates the impact of a six-month programme to address organizational stressors and promote staff-care and social support	Health determinants and risks; health system (workforce)	Not reported	Evaluation research/ Implementation research	Staff working for a psychosocial support organization	Non-government-controlled areas (Idlib)	Not reported
**Perkins (2018)** [60]	First: UK Last: Syria	First: academic Last: academic	Reports the incidence of psychological symptoms among school-aged children in Damascus and Lattakia	Health status	Not reported	Cross-sectional study	School children (8–15 years old)	Damascus, Lattakia	Not reported
**Rehman (2018)** [61]	First: Austria Last: Germany; Austria	First: academic Last: academic, clinical	Describes the leishmaniasis surveillance control program	Health status	November 2014– February 2016	Molecular–epidemiologic survey of cutaneous leishmaniasis in sentinel sites	General population (including IDPs)	Northern Syria (Aleppo, Idlib, Hama, Al-Raqqa, and Al-Hasakeh)	USAID & DFID
**Rodriguez-Llanes (2018)** [62]	First: Belgium Last: USA	First: academic Last: academic	Reports demographic characteristics of victims of chemical weapons attacks	War strategies / alleged IHL violations; mortality	March 2011–April 2017	VDC data analysis	Deceased victims of chemical weapons attacks	Non-government-controlled areas (Aleppo, Damascus, Hama, Idlib)	None
**Sawaf (2018)** [63]	First: Syria Last: Syria	First: academic Last: academic	Explores medical students’ attitudes and factors affecting their specialty choices and career plans	Health system (workforce)	August 2016	Self- administered questionnaire	Medical students at three universities in Damascus	Damascus	Syrian Private University
**Sikder (2018)** [64]	First: USA Last: Jordan	First: academic Last: independent	Assesses the effectiveness of a multilevel risk reduction intervention in maintaining Free Chlorine Residual in household drinking water	Humanitarian assessment, response or needs	January 2018	Cross-sectional study using interviews & observations; water quality testing	Chlorination station operators, well owners, households, Methology	Opposition-controlled areas in southern Syria (governorates not specified)	WoS (Amman hub) WASH Sector, UNICEF, UNICEF/NYC and Tufts University
**Sikder (2018)** [65]	First: USA Last: Jordan	First: academic Last: UN agency	Examines WASH access and needs in opposition controlled southern Syria	Humanitarian assessment, response or needs	June/July 2016–February 2017	Cross-sectional household surveys & water quality testing	Households	Opposition-controlled areas (Dara’a, Quneitra)	WoS (Amman hub) WASH Sector
**Turk (2018)** [66]	First: Syria Last: USA	First: academic Last: academic	Assesses medical students attitudes toward research	Health system (workforce)	Not reported	Self-administered questionnaire	Medical students at University of Damascus	Damascus	None
**Wong (2018)** [67]	First: USA Last: USA	First: academic Last: clinical	Reviews attacks on ambulances	War strategies / alleged IHL violations; health system (service provision)	January 2016–December 2017	Secondary data analysis on individual attacks reported by SNHR	NA	National	None
**Ahmad (2019)** [68]	First: UK Last: UK	First: academic Last: academic	Examines perceptions of married women living in low-income formal and informal neighborhoods in Aleppo on the effects of neighbourhood on their health and well-being	Health determinants and risks	April–June 2011	Semi-structured interviews	Married women living in informal and low-income formal neighbourhoods	Aleppo	European Commission FP7 programme grants MedCHAMPS and RESCAP-MED
**Abu Salem (2019)** [69]	First: Lebanon Last: Lebanon	First: academic Last: academic	Uses VDC data to confirm conflict events and identify fake news in FA-KES, a fake news dataset	Mortality	Not reported	Analysis of news articles and reports on incidence of deaths compared to VDC dataset	General population	National	American University of Beirut
**Alaryan (2019)** [70]	First: Syria Last: Syria	First: academic Last: academic	Reports on prescription drug misuse in Damascus and Damascus countryside during the conflict	Health system (medical products)	December 2016–March 2017	Cross-sectional survey	Community pharmacists	Damascus and Rural Damascus	Not reported
**Alhaffar (2019)** [71]	First: Syria Last: Syria	First: academic Last: clinical	Examines prevalence of burnout syndrome among resident physicians in training	Health system (workforce)	July 2018	Online questionnaire	Resident physicians in training from 12 hospitals in 8 governorates who spent at least one year in a residency program approved by the Syrian Commission of the Medical Specialties.	Damascus, Aleppo, Lattakia, Tartous, Dara’a, Rural Damascus, Hama, Homs	Not funded (self-funded)
**Alhaffar (2019)** [72]	First: Syria Last: Syria	First: academic Last: academic	Examines the prevalence of caries and oral health status among school children in Damascus, and associations with socio-economic status	Health status; Health determinants and risks	September–November 2017	School-based survey (questionnaire and clinical examination)	Seventh-grade school children in 10 randomly selected schools covering Damascus city	Damascus	Not funded (self-funded)
**Alhammoud (2019)** [73]	First: Qatar Last: UK	First: clinical Last: clinical	Reports the experience of one field hospital in Aleppo providing treatment of open shaft fractures	Health system (service provision)	July 2011–July 2016	Retrospective medical record review	Patients with open long bone fractures managed with external fixation	Aleppo	Not reported
**Alothman (2019)** [74]	First: Syria Last: India	First: academic Last: academic	Reports on war injury presentations to Hama National Hospital	Health system (service provision)	2017	Retrospective medical record review	War injured patients received by Hama National Hospital	Hama	Not reported
**Ballouk (2019)** [75]	First: Syria Last: Syria	First: academic Last: academic, government	Examines prevalence of dental caries among school-aged children in Damascus city	Health status	September 2016–January 2017.	School based oral health survey (clinical examination)	School children aged 8–12 years and resident in Damascus city during the study period	Damascus	Not funded (self-funded)^d^
**Blackwell (2019)** [76]	First: USA Last: not stated	First: humanitarian Last: humanitarian	Examines the impact of a cash-based assistance program on women’s empowerment and violence against women	Humanitarian assessment, response or needs; health status	March–August 2018	Semi-structured interviews	Woman aged 18–59 who received a cash payment over a three-month period	Al-Raqqa	UK Department for International Development (DFID)
**Douedari (2019)** [77]	First: UK Last: UK	First: academic Last: academic	Examines perceptions of local health providers and service users regarding health system governance, roles and relationships of institutional actors, and challenges and potential solutions	Health system (governance)	July–August 2016	Key informant interviews	Health system providers from health directorates, humanitarian NGOs, donors, and service-users	Opposition-controlled provinces (Aleppo, Dara’a, Hama, Idlib, Rural Damascus)	Chevening Scholarships
**Duclos (2019)** [78]	First: UK Last: UK	First: academic Last: academic	Examines cross-border humanitarian assistance and the challenges encountered by humanitarian health actors delivering health care in North-West Syria (Turkish border)	Humanitarian assessment, response or needs; health system (service provision)	September 2017	Mixed methods, including key informant interviews, desk reviews and expert consultations	Humanitarian aid professionals in Turkey-based organizations operating in North-West Syria, WHO-Turkey staff members and members of Syrian health directorates.	North-West Syria	London School of Hygiene and Tropical Medicine, Department of Infectious Disease Epidemiology, and the International Development Research Center (IDRC)
**Falb (2019)** [79]	First: USA Last: USA	First: humanitarian Last: humanitarian	Examines associations between depressive symptoms and their potential risk factors (including stressors, intimate partner violence)	Health status; health determinants and risks	March–April 2018	Cross-sectional survey	Married women who participated in a cash transfer program	Al-Raqqa	UK Department for International Development (DFID)
**Fardousi (2019)** [80]	First: Lebanon, UK Last: UK, Singapore	First: academic Last: academic	Examines perspectives of healthcare workers on issues of safety, resource management and handling of mass casualties during siege	Health system (workforce, service provision); war strategies / alleged IHL violations	Not stated	Key informant interviews	Syrian healthcare workers and service users who experienced siege in Aleppo in 2016 or Ghouta (Rural Damascus) in 2013–2017	Aleppo, Rural Damascus	Chevening Scholarships
**Fradejas-Garcia (2019)** [81]	First: Spain Last: N/A	First: academic	Explores experiences of remote cross-border operations	Humanitarian assessment, response or needs	Not reported	Ethnographic study through interviews	Aid workers and organizations – UN agencies, international NGOs and Syrian NGOs – providing relief assistance to Syria remotely	Turkish border city of Gaziantep	Not reported
**Hallak (2019)** [82]	First: Turkey Last: Turkey	First: academic Last: academic	Examines optimal shelter locations for IDPs	Humanitarian assessment, response or needs	December 2017–February 2018	Needs assessment using focus group discussions, key informant interviews and questionnaire with direct beneficiaries; mathematical modelling	Direct beneficiaries in 26 sub-districts within Idlib	Idlib	Not reported
**Hamid (2019)** [83]	First: Syria Last: Syria	First: academic Last: academic	Compares dental and gingival status among children with and without PTSD	Health status	Not reported	Case control study	Children (9–14 years old) who attended psychiatry department (cases) or dental service (controls)	Damascus	Damascus University
**Hamzeh (2019)** [84]	First: Syria Last: Syria	First:, academic Last: academic	Assesses knowledge and awareness of diabetes mellitus and its complications and effects of the conflict on care-seeking behavior among diabetes patients in Damascus	Health status; health determinants and risks	August–November 2017	Questionnaire	Diabetes mellitus patients attending clinics at the four main hospitals in Damascus	Damascus	Not funded
**Jamal (2019)** [85]^e^	First: UK Last: Lebanon	First: academic Last: academic	Examines the UNRWA health system and factors contributing to its resilience and enabling ongoing delivery of services to Palestinian refugees inside Syria during the crisis	Health system (service provision, governance, medical products, workforce)	February–August 2017	Key informant interviews, group model building sessions	UNRWA clinical and administrative professionals engaged in health service delivery for Palestinian refugees in Syria over the course of the conflict	Damascus, Jaramana (Rural Damascus), City Center Polyclinic^f^ and Aleppo	Wellcome Trust
**Mic (2019)** [86]	First: Turkey Last: Turkey	First: academic Last: academic	Examines optimal locations for primary health care centers in Idlib to allow services to the greatest number of people	Humanitarian assessment, response or needs	March–May 2018	Needs assessment using focus group discussions, key informant interviews and questionnaire with direct beneficiaries; mathematical modelling	Direct beneficiaries in 23 sub-districts within Idlib	Idlib	Çukurova University (Adana, Turkey)
**Mohammad (2019)** [87]	First: Syria Last: USA	First: academic Last: academic	Evaluates effectiveness of personalized supervision of residents on improving prescribing for asthma management, and effectiveness of mobile video training for inhaler technique	Health system (service provision)	April–May 2018	Clinical audits; evaluation of efficacy of video-mobile education	Asthma patients at internal medicine clinic in public hospital in Damascus	Damascus	Not reported
**Muhjazi (2019)** [88]	First: Egypt Last: UK	First: UN agency Last: humanitarian	Reports cutaneous leishmaniasis epidemiology over the course of the war	Health status; Health system (information system)	2007–2010, 2011–2018	Secondary data analysis of 1) MOH routine surveillance system, 2) EWARS, 3) MENTOR Initiative data	General population	National	Not funded
**Okeeffe (2019)** [89]	First: Syria Last: Netherlands	First: humanitarian Last: humanitarian	Describes blast-wound cases admitted to an MSF supported district hospital during the Raqqa military offensive and the first months of the post offensive period.	Health status; Health system (service provision); humanitarian assessment, response, and needs	June 2017–March 2018	Retrospective chart review	New blast-wound injured cases admitted to MSF-district hospital in Al-Raqqa in the offensive (June–October 2017) or post offensive period (October 2017–March 2018)	Al-Raqqa	Not funded
**Ri (2019)** [90]	First: USA Last: USA	First: academic Last: academic	Compares trends of attacks on healthcare and civilian casualites to assess feasibility of using publicly available data on attacks on healthcare facilities to describe population-level violence	Mortality; War strategies / alleged IHL violations	March 2011–November 2017	Secondary analysis of publicly available data from: VDC (civilian casualties), PHR (attacks on healthcare facilities), UNHCR (registered Syrian refugees)	Civilians	National	Not funded
**Roumieh (2019)** [91]	First: Syria Last: Syria	First: academic Last: academic	Assesses prevalence of postpartum depression among Syrian women in Damascus and examines associated risk factors	Health status; Health determinants and risks	January–December 2017	Cross-sectional questionnaire	Postpartum women attending 8 primary healthcare centers in Damascus	Damascus	Damascus University
**Terkawi (2019)** [92]	First: USA, Saudi Arabia Last: Saudi Arabia	First: humanitarian, academic, clinical, thinktank/ research organization Last: academic	Assesses disease burden and clinical presentations of children and adolescents attending a healthcare center in Atmeh district, Idlib	Health status	February–December 2017	Clinical data analysis	Children and adolescents attending healthcare center in Atmeh district	Idlib	Not funded
**Terkawi (2019)** [93]	First: USA, Saudi Arabia Last: Saudi Arabia	First: humanitarian, academic, clinical, thinktank/ research organization Last: academic	Examines health status and barriers to accessing antenatal care among pregnant women in Northwestern Syria	Health system (service provision); health status; health determinants and risks	February–December 2017	Medical record review; cross-sectional survey	Pregnant women attending healthcare centers in Atmeh district for antenatal care	Idlib	Not funded
**Vernier (2019)** [94]	First: Syria Last: UK	First: humanitarian Last: humanitarian	Assesses health status and mortality among IDPs who recently arrived to Ein Issa camp in Al-Raqqa	Health status; mortality; humanitarian assessment, response or needs; health determinants and risks	November 2017	Cross-sectional survey	IDPs who had arrived at Ein Issa camp since October 2017	Al-Raqqa	MSF
**Youssef (2019)** [95]	First: Syria Last: Lebanon	First: academic Last: academic	Examines the epidemiology of visceral and cutaneous leishmaniases in Lattakia prior to and during the conflict	Health status	2008–2016	Registry data analysis	General population	Lattakia	The American Society of Tropical Medicine and Hygiene (ASTMH) assisted with the publication expenses
**Ziveri (2019)** [96]	First: Belgium Last: Belgium	First: humanitarian Last: independent	Examines impact of a psychosocial support program on the well-being and agency of Syrian farmers receiving livelihood support	Humanitarian assessment, response or needs	April–August 2017	Randomized control trial among Syrian farmers	Farming households from ten randomly selected villages who fulfilled inclusion criteria	Not reported	Not funded

^a^The other governorates are not specified

^b^ *UOSSM* Union of Medical Care and Relief Organizations

^c^ Violation Documentation Centre (VDC), Syrian Center for Statistics and Research (CSR-SY), Syrian Network for Human Rights (SNHR), and Syria Shuhada website (SS)

^d^ Unfunded, the study was self-funded. This research was supported by Damascus University

^e^ first published online in 2019, hence captured in this dataset

^f^ The governorate is not specified

Health status is the most frequently researched theme, examined in 38 research papers covering nutrition [10, 94], communicable diseases and/or vaccination status [8, 18, 23, 26, 28, 30, 38, 41, 47, 54, 61, 88, 94, 95], mental health [20, 27, 39, 55, 56, 60, 79, 83, 91, 94], child [22, 41, 57, 92] and maternal [93] health, oral health [44, 72, 75, 83], gender-based violence [76], anaemia [43] and non-communicable diseases [39, 84, 94]. Of studies examining injuries, three are studies of hospital patients [29, 46, 89], one examines injury burden among children surveyed at home and in camps for internally displaced persons (IDPs) [41] and one reports injury counts among children and the general population as provided by key informants [32]. One additional study reports reasons for patient encounters at health facilities [28].

Of these health status studies, a few also report on socioeconomic associations with disease burden [43, 72, 79, 91], health seeking behaviours [84, 93] and exposure to violence as a determinant of health [94]. Several other papers focus primarily on health determinants and risks, including neighbourhood socioeconomic status [9, 68], occupational stress [59], food security [34], and smoking prevalence and smoking behaviours before and during the war [53].

Thirty-four research papers examine the various pillars of the health system. Research on *health workforce* includes studies of the prevalence of psychological symptoms and burnout among medical students and trainees [27, 71], workforce training [42], interventions using social media platforms as a teaching medium [12], consideration of the impact of conflict on workforce size, support or wellbeing [17, 25, 40, 49, 80, 85], including numbers of health workers killed or injured by attacks on health care [35, 36, 52], workforce wellbeing interventions [59], workforce requirements to address estimates of likely disease burden [20], and studies of medical student career plans [63] and attitudes to research [66].

*Health information systems* are studied largely in the context of communicable disease surveillance and comparison of surveillance systems covering government and non-government controlled areas [18, 23, 26, 88]. Two papers cover issues of health system *governance*, one through key informant interviews with health service providers, donors and end-users in opposition-controlled areas [77] and the other through interviews with UNRWA personnel that included consideration of adaptive mechanisms used to ensure resilience and ongoing function of the UNRWA health system [85]. *Medical products* are the focus of two papers, one of which surveyed community pharmacists in Damascus and Damascus countryside (Rural Damascus) regarding prescription drug misuse and characteristics of patients seeking such medications [70], and the other considered impacts of conflict on the UNRWA system, including on availability of medicines and medical supplies [85]. There are no studies on health *financing*.

Twenty-one papers cover issues of *service provision* including renal [17], mental health [20, 24, 56], orthopaedic [73], cancer [40], communicable disease surveillance [54], respiratory [87], antenatal [93], and trauma services [25, 74, 89], disruptions to service provision due to attacks on healthcare [35, 36, 49, 52, 67], challenges of service provision under siege [58, 80], factors enabling sustained UNRWA service delivery [85], and interplays of local service provision with cross-border humanitarian assistance [78].

Humanitarian assistance, response or needs (which included any studies conducted or analysis of services provided by humanitarian agencies) are the focus of 26 papers. These include estimates of IDP numbers and trends [14], humanitarian needs assessments among the general population, many of whom were displaced, in nine predominantly government-controlled governorates in 2014 [13, 14] and among the general population [48] and displaced and female-headed households in 10 largely urban government-controlled areas in 2016 [33]; identifying optimal locations for IDP shelters [82] and primary healthcare facilities [86] in Idlib based on beneficiary needs assessments and modelling; and a snapshot survey of community income and humanitarian assistance in Idlib [31]. Other studies included analysis of Qatar Red Crescent surveys of the impacts of the conflict on education, family and public health status [22] and diagnoses, injuries and comorbidities [41] among children in Northern Syria in 2015; and household surveys of water, sanitation and hygiene (WASH) and health outcomes in opposition-controlled Daraa and Quneitra in 2016–17 [65]. Review of humanitarian programmatic data and operations included middle-upper arm circumference screening, survey of living conditions and food security, and nutritional programming administered by Medecins Sans Frontiers (MSF) in Al-Raqqa in 2013 [10], MSF vaccine-preventable disease risk assessment, pre- and post-vaccine coverage surveys and immunization activity in Aleppo in 2015 [47], 2012–2014 surgical data from an MSF field hospital in Northwest Syria [19], blast injuries managed at an MSF-supported facility in Raqqa in 2017–18 [89], MSF paediatric consultations in Aleppo and Raqqa in 2013–16 [57], MSF assessment of health status of recently arrived IDPs in Al-Raqqa in 2017 [94], primary care services delivered by 10 Union of Medical Care and Relief Organisations (UOSSM) centres in opposition-controlled territories in 2014–2015 [28], and analysis of data from the humanitarian health response in contested and opposition-controlled areas in 2013–14 [32]. Additional interventions and program evaluations included delivery and evaluation of an intervention through provision of information and follow-up questionnaire in bread packages being distributed by a humanitarian organization in Northern Syria [21], evaluation of three modes of food assistance programming in Idlib in 2014–15 [34], evaluation of an International Rescue Committee cash assistance program on violence against women in Raqqa [76], evaluation of effectiveness of multi-level WASH risk reduction interventions in southern Syria in 2018 [64] and examination of the impact of a psychosocial support program on the wellbeing of a control and intervention group of farmers [96]. Several papers interviewed humanitarian workers, including humanitarian health staff working on non-communicable disease (NCD) care in Syria [50] and those involved in the cross-border humanitarian response from Turkey [78, 81].

Fourteen papers research health issues related to war strategies and alleged IHL violations, including an expert panel review of YouTube videos following a sarin gas attack [11] and interviews with healthcare workers in opposition-controlled areas regarding attacks on healthcare and challenges and experiences in responding to chemical attacks [49]. Other research in this theme examined attacks on health care [35, 36, 52, 67, 90], areas under or the effects of siege [40, 58, 80], and war-related mortality [15, 45, 51, 90] including a study of characteristics of deceased victims of a chemical weapons attack [62].

Mortality is the subject of ten papers, which report mortality counts provided by key informants in contested and opposition areas [32]; examine mortality data documented by the Violations Documentation Centre (VDC) [15, 51, 62]), examine associations between attacks on healthcare and civilian casualties [90] or confirm conflict events against war-related deaths from VDC in a fake-news dataset [69]; use capture-recapture methods on four datasets to estimate mortality in two governorates [16]; estimate the number of unique identifiable deaths by deduplicating four datasets [45]; use spatio-temporal death data to forecast conflict events [37] and report on a household survey of IDPs in Raqqa and retrospective one-year mortality, largely conflict-related deaths [94].

### Research themes by governorate

Themes studied vary by governorate (Table 1, Fig. 1). In Damascus, health status and the health system are the most frequently studied themes (*n* = 14 for each). The health system was also the main theme examined in studies of Aleppo (*n* = 12) and Idlib (*n* = 9). Humanitarian assistance, response or needs are most frequently studied in the north-west of Syria, including Aleppo (*n* = 8), Idlib (*n* = 7) and Lattakia (*n*  = 7), and of the studies examining specific governorates, all 14 governorates were covered in at least one paper. Of the papers examining war strategies and alleged IHL violations, the majority include a focus on Aleppo (*n* = 6) or Damascus (*n* = 5). On the national level, the health system is the most frequently studied theme (*n* = 8), followed by health status (*n* = 6), war strategies and alleged IHL violations (*n* = 4) and mortality (*n* = 3).

### Research themes by author country of affiliation

Themes examined vary by country of affiliation of authors (Table 1). Authors with Syrian affiliations commonly publish on health status (*n* = 21 for first authors, *n* = 15 for last authors), the health system (*n* = 12 for first authors, *n* = 7 for last authors), and health determinants and risks (*n* = 6 for first authors, *n* = 5 for last authors), while the most frequently researched themes among US-affiliated authors are the health system (*n* = 11 for first authors, *n* = 10 for last authors), humanitarian assistance, response or needs (*n* = 9 for first authors, *n* = 3 for last authors), health status (*n* = 8 for first authors, n = 3 for last authors) and war strategies and alleged IHL violations (n = 6 for first authors, n = 8 for last authors).

#### Field and operational activities publications

Table 3 presents a summary of the 31 papers reporting on field and operational activities, of which 12 describe humanitarian assessment, responses or needs, including development of a rapid gender analysis tool [127], cross-border, sectoral and cluster coordination mechanisms [104, 115–117], and needs assessments and/or operational programming [98, 105, 107–110, 122]. Nineteen papers discuss various aspects of the health system, most commonly reporting on experiences of establishing and / or presentations to field hospitals [99–101, 103], or establishing or delivering specific services including renal [102, 112], dental [106], mental health [113], obstetric [111], maternal and child health [119], tele-cardiology [120], tele-intensive care [114, 121], tele-radiology [118, 123] and polio outbreak response activities [124]. Other papers described the national tuberculosis control program [125], activities of Syrian expatriate medical associations in supporting the health system, including through training, establishment of hospitals and provision of telemedicine services [97], and translation and uptake of an online medical education platform into Arabic by Syrian medical students [126]. War strategies and alleged IHL violations are the secondary theme of two papers, one describing experiences in besieged settings [123] and one paper reporting birth outcomes by chemical weapons exposure status for pregnant women seen at Al Ghouta hospital in late 2014 [111]. Only 12 (39%) of these field and operational activities publications are first-authored by an author with a Syrian affiliation. Of the 21 publications with multiple authors, only 5 (24%) had a senior (last) author with a Syrian affiliation.

**Table 3:** Summary of conflict-related operational and organisational field experience publications, Syria January 2011–December 2019

First author, publication year	First & last (senior) authors’ country of institutional affiliation	Description	Theme (subtheme)^a^
**Hallam (2013)** [97]	First: UK Last: N/A	Lists some activities undertaken by Syrian expatriate medical associations, including provision of training, telemedicine consultations and establishment of hospitals	Health system (service provision, workforce)
**Harrison (2013)** [98]	First: Syria Last: Switzerland	Describes UNHCR’s mental health and psychosocial support program	Humanitarian assessment, response or needs
**Hasanin (2013)** [99]	First: Egypt Last: Egypt	Describes experiences establishing a field hospital in a district in Aleppo.	Health system (service provision)
**Sankari (2013)** [100]	First: USA Last: USA	Describes establishment and activities of field hospitals in Syria	Health system (service provision)
**Alahdab (2014)** [101]	First: Syria Last: USA	Describes the experience of field hospitals in Syria	Health system (service provision)
**Al-Makki (2014)** [102]	First: USA Last: USA	Describes experiences of provision of renal services and reports on establishment of the Syrian National Kidney Foundation by two Syrian American nephrologists	Health system (service provision)
**Attar (2014)** [103]	First: USA Last: N/A	Reports number and type of presentations to four field hospitals in Aleppo during a two week period in December 2013	Health system (service provision)
**Dolan (2014)** [104]	First: Not mentioned Last: N/A	Describes the cross-border nutrition coordination experience in southern Turkey	Humanitarian assessment, response or needs
**Egendal (2014)** [105]	First: Syria Last: Syria	Describes the World Food Programme’s emergency programme in Syria	Humanitarian assessment, response or needs
**Joury (2014)** [106]	First: Syria Last: N/A	Describes a Syrian community-based outreach dental education project named “Syrian Smiles” that aimed to provide dental care services and improve knowledge, skills and attitudes of dental students	Health system (workforce, service provision)
**Khudari (2014)** [107]	First: Syria Last: Syria	Describes WHO’s nutrition activities in Syria	Humanitarian assessment, response or needs
**Kingori (2014)** [108]	First: Jordan Last: Syria	Describes the nutrition crisis response in Syria and UNICEF’s nutrition activities	Humanitarian assessment, response or needs
**Littledike (2014)** [109]	First: Syria Last: Not clear	Describes World Vision International’s experiences supporting nutrition and primary healthcare programming to IDPs in Aleppo in 2013–2014	Humanitarian assessment, response or needs
**Reed (2014)** [110]	First: Syria Last: N/A	Describes needs assessment and food and voucher assistance program implemented by GOAL in northern Syria	Humanitarian assessment, response or needs
**Hakeem (2015)** [111]	First: Syria Last: Syria	Reviews medical records of 211 pregnant women seen at Al Ghouta hospital in Sept-Nov 2014 (following August 2013 chemical attack) and reports birth outcomes by self-reported chemical exposure status	Health system (service provision); health status; war strategies / alleged IHL violations
**Saeed (2015)** [112]	First: Syria Last: N/A	Describes the number of renal transplant centers in Syria, their staffing and activity during the war	Health system (service provision)
**Jefee-Bahloul (2016)** [113]	First: USA Last: Turkey	Describes development and application of a tele-mental health system	Health system (service provision)
**Moughrabieh (2016)** [114]	First: USA Last: USA	Describes a remote tele-intensive care service and training of supporting staff in Syria	Health system (service provision)
**Abdulahi (2017)** [115]	First: Syria Last: N/A	Describes Nutrition Sector co-ordination mechanisms implemented in Syria since 2013	Humanitarian assessment, response or needs
**Abdullah (2017)** [116]	First: Syria Last: Jordan	Describes the Whole of Syria Nutrition Coordination mechanisms implemented in 2015	Humanitarian assessment, response or needs
**Madanİ (2017)** [117]	First: Turkey Last: N/A	Describes the nutrition response in Syria including activities of the Nutrition Cluster	Humanitarian assessment, response or needs
**Mohammad (2017)** [118]	First: Saudi Arabia Last: Syria	Descrbes the Teleradiology Relief Group including support provided to a facility in eastern Ghouta	Health system (service provision)
**Aburas (2018)** [119]	First: USA Last: USA	Describes a local medical humanitarian initiative (the Brotherhood Medical Centre), established to provide free, accessible maternal and child health services to Syrian IDPs in Atimah, Idlib.	Health system (service provision)
**Alrifai (2018)** [120]	First: USA Last: USA	Describes tele-cardiology service provision from the United States to support hospitals in Syria	Health system (service provision)
**Ghbeis (2018)** [121]	First: USA Last: USA	Describes implementation of the “Tele-Pediatric Intensive Care” program	Health system (service provision)
**Lester (2018)** [122]	First: UK Last: N/A	Describes a training programme for White Helmets members in which the trauma-informed framework was used to design and implement a range of mental health and psychosocial interventions.	Humanitarian assessment, response or needs
**Masrani (2018)** [123]	First: USA Last: Saudi Arabia	Describes a remote tele-radiology service supporting staff in besieged Ghouta between 2015 and 2018	Health system (service provision); War strategies / alleged IHL violations
**Mbaeyi (2018)** [124]	First: USA Last: Switzerland	Describes the WHO response to the emergence of vaccine-derived polioviruses (VDPVs) in Deir ez-Zor governorate in Syria in 2017	Health status, Health system (service provision)
**Muhjazi (2018)** [125]	First: not stated Last: not stated	Describes the national tuberculosis control program	Health system (service provision)
**Kouba (2019)** [126]	First: Syria Last: USA	Describes translation of an online US-based medical education platform from English into Arabic by Syrian medical students, and uptake of the platform by Syrian universities and students	Health system (workforce)
**Quay (2019)** [127]	First: Switzerland Last: N/A	Describes development of Rapid Gender Analysis by CARE during its humanitarian response in Syria	Humanitarian assessment, response or needs

^a^Field hospitals are categorized under the health system theme as although these hospitals may be funded by humanitarian agencies and NGOs, the staff are generally local healthcare workers

#### Narrative personal reflections

Eight publications were reflections or first-hand personal accounts of experiences inside Syria. These included reflections of an expatriate physician on missions to field hospitals [128] including in Aleppo in 2013 [129]; experiences of a resident physician working in the neurosurgery department of a hospital under siege in Aleppo [130]; experiences of a medical resident working in Aleppo University Hospital following a chemical weapons attack, including reports of number of patient presentations and outcomes [131]; experiences of medical volunteers during a mission in Idlib [132]; and personal and professional experiences of a Syrian neurosurgeon working in Syria [133]. Two papers are experiences of medical students, one reporting experiences undertaking research and accessing research training [134] and another reporting experiences of studying medicine in conflict [135].

#### Challenges reported in researching Syria

Researchers note a range of considerations and challenges of conducting research in/on Syria (Table 4). *Contextual* challenges are largely related to safety and security issues, including the impact of conflict on access, often being limited to specific governorates or relatively safer areas, and conflict events impacting the ability to conduct research as planned.

**Table 4:** Selected considerations and challenges of conducting conflict-related health research in and on Syria as described in the literature, January 2011- December 2019

CONTEXTUAL CHALLENGES (include issues of safety, accessibility, stakeholder engagement & cultural considerations)	METHODOLOGICAL	ADMINISTRATIVE (include issues related to research permits and permissions, logistics, research capacity)
***Conflict context and safety and security considerations*** • impacting on accessibility [13, 14, 16, 22, 25, 33, 41, 70, 71]. • impacting selection of school study sites [60] • impacting ground team composition and generating reliance on local staff [21, 47, 61) • impacting ability to conduct research as planned, including delaying data collection [34]; requiring short data collection periods or terminating fieldwork earlier than planned [9, 68]; postponing fieldwork [85]; requiring timing and setting of interviews that were often not conducive to research [9]; and impacting data collection [17] ***Ethical considerations due to the context*** • Assuring anonymization of data and / or locations, including for data integrity and participant / facility security [25, 21] • Not including community representatives and other stakeholders in the study due to ethical concerns regarding security, anonymity and any potential risks of coercion regarding aid and services [85] • Study design considerations, including not using a control group in a clinical study of PTSD treatment in order to benefit as many as possible during wartime [56], and delivering a psychosocial intervention to the control group after completion of data collection [96] ***Researcher identity and relationship to the context*** • Wariness of researchers affiliated with western institutions [9, 68]	***Participant recruitment, sample size*** Conflict context impacting availability of research subjects: • Conflict context potentially impacting willingness to participate [21, 53, 77, 80] and requiring informal approaches to participants through trusted colleagues [9, 68]. • Participant recruitment challenged by confidentiality and safety considerations, limited electricity / internet access and time constraints [77, 80] • Limited availability of research participants in studies of /with health workers due to the need for extra emergency service provision during times of conflict [42] or due to facilities being closed or out of service [70] • Practical and logistic difficulties due to war making it difficult to increase number or age range of participants in a clinical study [55] • Few witnesses left, may be unwilling or unsafe to report what they have seen, or might not know how to report [16] ***Study design*** • No appropriate control group who are unexposed to war, preventing examination of associations of disease burden with war [41] • Challenges evaluating the outcome of interventions in conflict settings [21, 34] • Sampling frame considerations due to limited population data and ongoing population displacement [33] ***Data collection*** • Need for data collection to be conducted remotely [17, 80]; conducting interviews remotely may impact rapport, non-verbal cues; generates additional ethical considerations of using third-party software [77] • Lack of / limited follow-up including due to accessibility and safety [43, 73] • Disruption / breakdown of health surveillance and data infrastructures [23, 54] ***Potential research biases*** • Potential for social desirability and cultural familiarity biases when interviewers are diaspora / refugees from same context [80] • Potential for recall and reporting biases, including possible over-reporting among survey respondents if perceived that responses might influence access to aid and services [79] [94], possible under-reporting or lack of willingness to speak openly including due to fear and security considerations [22, 41, 50, 51, 94] • Potential reporting biases in studies of attacks on healthcare e.g. attacks on larger facilities may be reported more than those on smaller ones if there is less impact or fewer staff witness it; political agendas of witnesses and those reporting on attacks might influence reporting; restricted access to verify reports [35, 52, 67] • Potential survival bias in retrospective mortality surveys e.g. if all members of a household were killed, will not be captured in household level surveys where participants asked about household deaths [94] • Selection and respondent biases impacting representativeness; limited generalizability due to restricted geographic access and because not all governorates impacted by war to the same extent and in the same way [27, 33, 48, 49] • Potential for data collection and categorization to be biased by motivations of researchers, political or advocacy groups [51] ***Data availability and quality*** • Missing data and limited data availability [20, 28, 74] • Impact on data quality and availability due to patient care taking precedence over documentation during crisis [89] • Difficulties of ascertaining casualty counts in an active conflict [90] • Extracting additional data on war injuries limited as names were deleted from paper medical records for security reasons [74] • Difficulties with outcome measurement and follow up data, including inability to return for follow up because of security issues, inability to follow up by phone because of disrupted phone network [19] • Difficulties ascertaining response rate due to inability to contact participants directly, reliance on third party [24] • Absence of / difficulties with establishing population denominator [32, 38, 57, 61] • Inability to validate authenticity of data collected from online video sources [11]	***Ethics / Institutional Review Board processes*** • Lack of formal local ethics committees and governance processes [59, 61, 80] • Concerns with obtaining written consent related to anonymity and potential risks associated with participation [61] ***Permissions and permits*** • Need to secure specific travel permissions for local participants to attend workshops outside Syria [85] • Permission to conduct assessments sought from community leaders as needed [13, 14, 33, 34, 48] ***Local contextual factors*** • High turnover of staff including in government departments meant limited engagement and inability to effectively translate research findings into policy and practice [9] • Conflict impacting availability of research funding [53]

Researchers report numerous *methodological* challenges, including limited data availability and access, data quality and completeness concerns, lack of population denominator data, and potential for bias at all stages of the research process.

*Administrative* considerations are largely related to securing permissions to conduct research and ethical review board processes, most notably the absence of such formal in-country governance structures. Conflict also impacted other aspects of research administration, including the availability of research funding.

## Discussion

Although increasing over time, our analysis shows that a relatively limited number of research studies focused on health or populations inside Syria have been published over the course of the conflict to 2019. There are several geographic and thematic information gaps which likely reflect a myriad of factors, including issues of access and other challenges of conducting research during active conflict. There is a relative paucity of research on several key pillars of the health system such as governance, financing and medical products, and on a number of critical population health issues such as NCDs. Research coverage of governorates shows a heavy emphasis on Damascus and the north-west, with comparatively little research covering the north-east and the south of Syria. Our analysis does not identify any research on some specific vulnerable populations such as those currently or formerly imprisoned or detainees, and very few studies of besieged areas.

### Some thematic areas have received limited research attention

Health status is the most frequently researched theme, with communicable diseases the most common focus. Given the disruption of health and social infrastructures during conflict, with attendant impacts on communicable disease surveillance and control, and the global health threat posed by outbreaks of communicable diseases such as polio, it is not surprising that communicable disease epidemiology and control is a major focus of research attention. Similarly, mental health is the focus of several papers which cover a number of population subgroups, including medical students [27], women [79, 91], children [56, 60, 83], selected patient populations [55], and IDPs [39, 94], in addition to modelling estimates of disease burden and associated workforce requirements for the general population [20]. This is a crucial area of research in a conflict context and in a cultural setting where mental health issues remain stigmatized.

Other direct health effects of conflict receive comparatively little research attention. For example, injury epidemiology is covered in just five papers, three of which described a cohort of inpatients with abdominal [29], paediatric chest [46] or blast injuries [89]. Understanding injury epidemiology is critical to informing health and social system needs, both to address immediate acute care needs but also to inform estimates of likely disability burden in future and requirements for health and social infrastructures. This limited research focus may reflect data gaps due to lack of research infrastructure and/or prioritization of lifesaving provision of care above record-keeping in acute emergencies and mass casualty events. The referral of many wounded individuals for treatment in neighboring countries may have also contributed to this gap, supported by an observation from our broader scoping review that there is more injury research from Turkey, Israel and Jordan than there is from within Syria.

NCDs are another major research gap, despite being identified as such in 2015 and a call for action issued [136]. Prior to conflict onset, Syria was advancing in its demographic and epidemiological transition, with NCDs dominating disease burden and health expenditure [137]. Left unaddressed and due to disruptions to the health system and altered health behaviours and preventive measures, this underlying disease burden is likely to have magnified. Studies among Syrian refugees indicate a high NCD burden [138]; a similar high burden likely exists among populations inside Syria. This warrants urgent research attention.

For a protracted conflict that has caused a large number of deaths, surprisingly few research studies have examined mortality. Notably, most of the mortality research considers direct war-related deaths. Few studies report on general mortality or examine indirect conflict-related deaths, such as those due to treatment forgone and service unavailability, for instance cancer deaths due to lack of access to chemotherapy or end-stage renal failure deaths due to lack of dialysis services (one paper provides an anecdotal estimate of renal deaths for one centre [17]). Such information is essential at the facility, governorate and national levels, both to inform immediate needs and to drive accountability – the effects of war, and the impacts of atrocities such as killing a doctor or attacking a hospital, extend far beyond the immediate acute losses.

Accurately estimating mortality in conflict presents numerous challenges [16] and several factors may explain the relative paucity of mortality research in Syria. Human rights organizations tracking deaths, such as VDC, are reliant on ground reporters, but relative inaccessibility of some geographic areas influences data collection and completeness. Local researchers based inside Syria may find it difficult to investigate this sensitive issue. Insecurities may render conducting household assessment on representative samples difficult, or generate security threats to families, witnesses or data collectors. In some instances, there may be no witnesses left to report the deaths [16]. Disruption of health information infrastructure means limited availability and completeness of data from official local sources such as hospital records and vital registration data. The fact that the United Nations stopped officially reporting deaths in Syria since 2014 introduced a further barrier to rigorous mortality analyses. Mortality data are also highly politicised. Recognising the data limitations and challenges, there is a clear need to advance methods for researching mortality in conflict, including developing better estimates of mortality considering the difficulty in enumerating deaths (numerator) in a highly charged political environment and the changing population numbers (denominator) due to displacement.

War strategies and IHL violations are the focus of several papers, including those examining attacks on health care, effects of siege, and chemical weapons attacks. Attacks on health care in Syria have been well documented, both by the research community as well as by a number of human rights and humanitarian organisations. There are numerous methodological and contextual challenges to such research. Issues noted among the studies in this review included concerns about validation of reported data on either chemical weapons or attacks on healthcare infrastructure and personnel [11, 35, 52, 67]. Reporting commonly depends on ground reporting systems which are subject to their own inherent limitations [35, 67].

The limited research on key health system pillars, namely governance, financing and medical products, is also of concern, particularly as governance and financing are critical to any consideration and planning for health system rebuilding.

### There is geographic variation in research volume and thematic issues examined

Focus of research covering areas such as Damascus, a Syrian government stronghold, is heavily on population health status and the health system, while research on northwest Syria (Aleppo and Idlib) focused more on the health system, particularly attacks on healthcare and other IHL violations, and humanitarian needs and assistance. Research on northeast Syria is extremely limited, and no research specifically on non-government controlled areas controlled by IS was identified in this review. These findings likely reflect security and access issues, in addition to conflict events and operational needs on the ground. For example, international agencies and humanitarian organizations based in Turkey have access to the opposition-controlled areas of north/northwest Syria, directly or through Syrian/diasporic health and humanitarian organizations, including through cross-border assistance operations. This may facilitate access to populations and humanitarian providers and collection of data.

It is important to note that not all geographic areas of Syria have been impacted to the same extent and in the same ways by the conflict. Further, prior to the conflict there was considerable geographic variation in Syria’s health system infrastructures, workforce distribution and resourcing [139], and demographic and population health profiles also varied across the country. Current population and humanitarian health issues and research needs therefore likely vary across Syria; such variation is not captured in this quantitative thematic assessment of number of research studies conducted per governorate. Future research should seek to identify the specific health research needs within and between governorates.

### Vulnerable groups and hard-to-reach populations receive limited research attention

There is limited research on some specific population subgroups. The protracted conflict has resulted in chronic and large-scale displacement inside Syria, with over 6 million IDPs [140]. New displacements are still occurring. It is estimated that 2.5 million Syrians were subjected to siege at some point during the conflict [141]. It is reported that to March 2021, more than 149,000 Syrians had been detained or forcibly disappeared [142]. These highly vulnerable populations are rarely studied. For example, only three papers [40, 58, 80], one field and operational activities publication [123] and one personal narrative [130] covered issues of siege. Understanding the health status and humanitarian needs of such populations is essential in order to inform programmatic action.

### Novel methodologies have been applied to study health in an active conflict zone

Numerous challenges are reported in the conduct of this body of research, largely consistent with those described by researchers who have worked in and studied a range of other active conflict zones. These include issues of access, data quality, quantity and availability (including lack of denominator data due to ongoing population displacement), and security considerations, including impacts on participant willingness to engage. A number of novel tools and methodologies were developed and described in this literature in order to overcome research constraints and to study conflict-related health issues. Humanitarian interventions have been used to piggy-back health messaging and research. For instance, El-Khani et al. distributed parenting psychosocial support information and questionnaires in humanitarian bread deliveries [21], highlighting the scope for existing humanitarian routes to both distribute information and serve as a research platform. Social media is increasingly used as a research tool, including in conflict settings. Research included in this review has used social media in a number of ways, including as a data collection tool [8, 11] and as a platform for delivery of an educational intervention [12]. Communication technologies have also been incorporated into research approaches, including through use of services such as WhatsApp as part of a tool to monitor attacks on healthcare in Syria [35], and secure messaging platforms used for remote interviews and data collection, including to overcome security considerations and access constraints [49, 77, 80].

### Strengthening academic and operational research collaboration is important

For the majority of research publications, the institutional affiliations of first and/or last authors are academic (universities or university hospitals). A limited number are authored by individuals with a humanitarian organizational affiliation. Only a few papers had a mix of first/last authors from both academic and humanitarian organisations. Humanitarian organisations have a critical role to play in conducting policy- and practice-oriented research [143]. Strengthening academic-operational research collaboration is important, including for issues of data access and data sharing. For instance, much service provision data, however (in)complete, is held by humanitarian organizations, international agencies and government bodies. Sharing of information with researchers for secondary data analysis is often a fraught process. Bridging this information divide and encouraging collaboration between researchers and operational organisations might minimize duplication of effort, increase relevance of the research to the endusers and help ensure best outcomes for the populations who must be the ultimate beneficiaries of research. Humanitarian agencies also often have readier access to study populations than do academics. Academic researchers may face additional barriers to timely information dissemination; even when data are available to researchers, lengthy institutional processes, research governance mechanisms, protracted times to data access and peer-reviewed publication times may mean that data cannot actually be used and published in a timely manner to inform operational and policy activity. Academic-humanitarian research partnerships may help address such barriers. For example, from the Syrian refugee context in Lebanon, an academic-humanitarian-public health authority research collaboration utilizing the complementary skills and expertise of each partner has ensured a successful and comprehensive project approach [144].

In this study we also identified a number of non-research publications reporting on operational and field activities and personal reflections. Such sharing of experiences should be encouraged, including by journals and journal editors.

### Efforts to mitigate potential barriers to Syrian-led research in and on Syria warrant attention

This review shows good representation of first and last authors from Syrian institutions, with approximately a third of first authors and almost a quarter of last authors having Syrian institutional affiliations. This does not reflect the total number of first and last authors from Syria or with Syrian background, as conflict-related displacement of Syrian academics means that some are publishing but are now affiliated with institutions in countries outside Syria.

There are several potential barriers to Syrian researcher engagement which warrant attention. These include general difficulties in access and insecurities, limited supply and/or capacity of Syrian researchers based inside Syria, and disruption or collapse of research infrastructure in Syrian institutions, including through recurrent attacks on universities, and widespread oppression of academics and freedom of speech more broadly which have had a detrimental impact on higher education institutions in Syria [145, 146]. Additionally, capacity building and training opportunities are limited: one study in this review reports lack of adequate training, research facilities and mentorship as barriers to research among undergraduates [66] and others also describe additional resource and funding constraints [134].

Such potential barriers to local research production must be addressed. Utilization of various techniques, e.g. use of pseudonyms to address security concerns, and implementation of contextually appropriate capacity building initiatives and frameworks, such as that proposed by others [147], to support more Syrian researchers and Syrian institutions to lead research on the health issues affecting their country and its people, is warranted.

### Strengths and limitations

To the best of our knowledge, this is the first review to characterize the body of published research indexed in the main citation databases and examining health inside Syria over the course of the conflict. Themes assigned reflect a paper’s major focus and do not necessarily capture all issues covered in a given publication. Assignment of key themes is necessarily subjective, and some papers may cover multiple thematic areas. Some of the themes are not mutually exclusive but were presented in distinct categories to highlight conflict-related gaps. For example, health-related IHL violations such as attacks on healthcare are determinants of health but specific to conflict settings; we therefore considered them in a separate category to the ‘health determinants and risks’ category, which captures traditional behavioural, physiological, environmental and social determinants of health that are also present in non-conflict zones. The scope of this paper is restricted to population and humanitarian health and health systems research, so studies of war strategies and IHL violations are limited to health-related violations and do not include publications examining attacks on non-health civilian infrastructures such as schools and bakeries, which are also upstream determinants of health.

Notably, this quantitative assessment of number of research studies conducted per governorate does not take into account research quality – a single high quality study may provide greater and more useful information than multiple lower quality and less rigorous publications.

Our summary of key challenges described in the papers is not an exhaustive list of all limitations noted by the authors but rather captures the main and frequently described issues. Relevant studies may have been excluded, or missed if published in journals not indexed in the seven bibliographic databases searched. For example, Arabic language papers published in local or regional journals are not captured in this review. Our search did not include the grey literature, so the findings of this review likely to do not fully capture all operational and field research undertaken and published by humanitarian organisations. Additionally, research may be undertaken to inform policymaking or programmatic action but not published. Finally, academic research is often subject to a publication lag, so it is possible that some of the information gaps identified in this review are the subject of current work and forthcoming research publications.

## Conclusions

Whilst there is a growing body of research examining population health issues inside conflict-ravaged Syria, there are considerable geographic and thematic gaps, and issues and populations that warrant focused research attention. Recognising the myriad of complexities of researching active conflict zones, including issues of data completeness, coverage and politicisation, it is essential that research in and on Syria continues, in order to build the evidence base and inform policy and programmatic actions that are required to protect and promote health in Syria following a decade of conflict.

## Supplementary Information

**Additional file 1.** Literature Search strategy for the full scoping review.

## Data Availability

Research data and material are available for peer review.
