# Supplementary material for: Conflict-related health research in Syria, 2011–2019: a scoping review for The Lancet - AUB Commission on Syria
Source: Confl Health. 2021 Dec 14;15:92. doi: 10.1186/s13031-021-00384-3 (PMC8672497; doi:10.1186/s13031-021-00384-3)
Supplement: Supplementary file 1 — Additional file 1. Literature Search strategy for the full scoping review. [file 13031_2021_384_MOESM1_ESM.docx]

**Conflict-related health research in Syria, 2011-2019: A scoping review for *The Lancet* - AUB Commission on Syria**

**Supplementary material**

**literature Search strategy for the full scoping review**

| Database | Search terms in Title/Abstract  (added till February 10^th^, 2020) | No. of Records |
| --- | --- | --- |
| [PubMed](https://www-ncbi-nlm-nih-gov.ezproxy.aub.edu.lb/pubmed) | (((syria[Title/Abstract]) OR syrian*[Title/Abstract]) NOT hamster*[Title/Abstract]) AND ("2011"[Date - Publication] : "2019"[Date - Publication]) | 2,059 |
| [Medline (OVID)](http://ovidsp.tx.ovid.com.ezproxy.aub.edu.lb/sp-3.32.0a/ovidweb.cgi)  Ovid MEDLINE(R) 1946 to July Week 1 2019 | 1 ((syria or syrian*) not hamster*).ab,ti.  2 limit 1 to yr="2011 – 2019" | 1,257 |
| [CINAHL Complete](http://web.b.ebscohost.com.ezproxy.aub.edu.lb/ehost/search/basic?vid=0&sid=158b37e9-a216-4167-8ede-5c807b108e48%40pdc-v-sessmgr03) | TI ( ((syria or syrian or syrians) not (hamster or hamsters)) AND PY 2011 - 2018 ) OR AB ( ((syria or syrian or syrians) not (hamster or hamsters)) AND PY 2011 – 2019 ) | 1,003 |
| [Global Health](https://www-cabdirect-org.ezproxy.aub.edu.lb/cabdirect/search)  (Your product: Global Health) | title:(syria OR syrian* NOT hamster*) OR ab:(syria OR syrian* NOT hamster*) AND yr:[2011 TO 2019] | 1,104 |
| [EMBASE](https://www-embase-com.ezproxy.aub.edu.lb/#search) | ('syria':ab,ti OR 'syrian*':ab,ti) NOT 'hamster*':ab,ti AND [2011-2019]/py | 2,592 |
| [Web of Science](http://apps.webofknowledge.com.ezproxy.aub.edu.lb/WOS_GeneralSearch_input.do?product=WOS&search_mode=GeneralSearch&SID=E4kXMkeZfGVNK5LewgI&preferencesSaved=) | TS=(SYRIA OR SYRIAN*) NOT TS=HAMSTER*  Time span = 2011- 2019  Science Citation Index Expanded (SCI-EXPANDED) --1900-present | 3,470 |
|  | TS=(SYRIA OR SYRIAN*) NOT TS=HAMSTER*  Social Sciences Citation Index (SSCI) --1900-present | 2,075 |
|  | TS=(SYRIA OR SYRIAN*) NOT TS=HAMSTER*  Arts & Humanities Citation Index (A&HCI) --1975-present | 1,303 |
| [Scopus](https://www-scopus-com.ezproxy.aub.edu.lb/search/form.uri?display=basic) | (TITLE ( syria ) OR TITLE ( syrian* ) OR ABS ( syria ) OR ABS ( syrian* ) AND NOT TITLE ( hamster* ) AND NOT ABS ( hamster* ) ) AND PUBYEAR > 2010 AND PUBYEAR < 2019 | 9,933 |
| Total | | 24,814 |
| Additional records identified through other sources | | 311 |
| Total (*de-duplicated based on automated matched Title & Year in Endnote. Author names checked manually*) | | 13,699 |
